# *In-vivo* anterior segment OCT imaging provides unique insight into cerulean blue-dot opacities and cataracts in Down syndrome

**DOI:** 10.1038/s41598-020-66642-1

**Published:** 2020-06-22

**Authors:** Julie-Anne Little, Aman-Deep S. Mahil, Patrick Richardson, J. Margaret Woodhouse, Valldeflors Vinuela-Navarro, Kathryn J. Saunders

**Affiliations:** 1 Centre for Optometry and Vision Science, Biomedical Sciences Research Institute, Ulster University, Coleraine, United Kingdom; 2 School of Optometry and Vision Sciences, Ulster University, Cardiff, United Kingdom; 3 Aston Optometry School, Aston University, Birmingham, United Kingdom

**Keywords:** Biomarkers, Eye manifestations, Lens diseases

## Abstract

Down syndrome (DS) is frequently associated with cataract, but there remains scant information about DS cataract morphology. Supra-nuclear cataracts in DS have been proposed as indicative of beta-amyloid (Aβ) aggregation and thus potential biomarkers for Alzheimer’s (AD). This study employed anterior segment OCT (AS-OCT) and slit-lamp (SL) photography to image the crystalline lens in DS, compared with adult controls. Lens images were obtained post-dilation. Using MATLAB, AS-OCT images were analysed and lens opacities calculated as pixel intensity and area ratios. SL images were classified using LOCS III. Subjects were n = 28 DS (mean ± SD 24.1 ± 14.3years), and n = 36 controls (54.0 ± 3.4years). For the DS group, AS-OCT imaging revealed the frequent presence of small dot opacities (27 eyes, 50%) in the cortex and nucleus of the lens, covering an area ranging from 0.2–14%. There was no relation with age or visual acuity and these dot opacities (p > 0.5) and they were not present in any control lenses. However, their location and morphology does not coincide with previous reports linking these opacities with Aβ accumulation and AD. Four participants (14%) in the DS group had clinically significant age-related cataracts, but there was no evidence of early onset of age-related cataracts in DS.

## Introduction

Down syndrome, caused by a full or partial copy of the 21^st^ chromosome, is the most common chromosomal aneuploidy in humans^1^. Individuals with DS have an increased prevalence of ocular sequalae, including refractive errors, poor vision, strabismus, keratoconus and cataract^2–13^. Life expectancy for those with Down syndrome has significantly increased in recent decades, and thus management of age-related chronic healthcare conditions is ever more important^14^. Cataract is the most common age-related ocular disease, and is reported with increased frequency in DS. Prevalence rates for cataract in DS vary greatly from 4–72% depending on the profile of the cohort assessed^8–13^. Previous reports have often recruited participants from institutionalised or clinical populations and have utilised a variety of methods to examine the crystalline lens; ranging from a cursory, undilated examination to more in-depth mydriatic assessments. The age of participants has also varied across studies and there is scant published information describing the features of cataract found in Down syndrome, particularly the aetiology and type of cataracts. Congenital cataract is reported in 1.4% cases with DS^15^, while other reports identify cataracts similar to those seen in the ageing eye.

Due to the triplicate of chromosome 21 and subsequent overexpression of the amyloid precursor protein, Down syndrome is associated with increased risk of Alzheimer’s disease (AD). Senile Aβ plaques and neurofibrillary tangles in the brain of DS adults aged 35 to 40 years and older have been reported^16,17^. Zana *et al*.^16^ report the rates of dementia in Down syndrome at 8%, 55% and 75% in those aged 35–49, 50–59 and over 60 years respectively. Clinical diagnosis of AD often relies on communication and an assessment of a patient’s ability to perform cognitive tests. Thus, while AD diagnosis is complex in typically developed adults, it poses even greater challenges in those with a learning disability. There is a need for alternative, objective and non-invasive methods of detecting the presence of AD. It has been proposed that supra-nuclear cataracts in DS are indicative of beta-amyloid aggregation and thus could be a biomarker for Alzheimer’s disease^18,19^. However, given the minimal information currently available regarding the morphology and frequency of cataract in DS, particularly in non-clinical DS populations, a better understanding of its prevalence, severity and morphology is required.

Slit lamp examination is the conventional method used to examine cataract and SL photography enables photograding of images. LOCS III is the most commonly used classification system for age-related cataract and requires optic section and retro-illumined images of the crystalline lens. However, anterior segment ocular coherence tomography (AS-OCT) is emerging as an alternative means of examining cataract. This technology uses infra-red light to deliver cross-sectional structural imaging of the lens, and lens opacities appear as hyper-reflective surfaces^20^. Good agreement has recently been reported between traditional methods of visualising cataractous changes and OCT imaging of the lens. AS-OCT also offers the opportunity to accurately examine the structure of the lens, as, unlike photographic optic sections on SL, where the angle of observation can vary, AS-OCT imaging is centred on the visual axis. AS-OCT also allows quantification of the location and extent of lens opacities through image analysis^21,22^.

The present study will investigate the morphology of cataract in a non-ophthalmological sample of participants with DS, and explore the feasibility of utilising SL examination, SL photography and AS-OCT imaging for examining the DS lens.

## Results

From the 30 participants recruited with DS, two participants were excluded during preliminary testing: one due to a recent brain tumour diagnosis and the other because it was not possible to position their wheelchair appropriately for imaging assessment. Attempts were made to image both eyes of all participants, however, in two participants one eye was excluded due to the presence of a uniocular corneal degenerative disease with neovascularisation, and a significant esotropia with poor fixation. Accordingly, SL examination and imaging of 54 eyes from 28 participants with DS were conducted. The mean age of participants was 24.10 ± 14.20 (standard deviation, SD) with a range of 6 to 55 years, and 17 participants were male. Dilated pupil diameter was measured at 8–9 mm for all participants.

SL examination was successfully completed in 96% (n = 52) DS and 100% of control eyes. Optic section SL imaging was successfully obtained in 39 (72%) DS and all control eyes. Retroillumination SL images were more difficult to capture in the DS group and the success rate dropped to 28% (n = 15). AS-OCT images were obtained from 33 (61%) DS and 46 (82%) control eyes. Table 1 presents data from LOCS III photograding, AS-OCT image analysis, and visual acuity of participants.

**Table 1:** LOCS III grading and AS-OCT quantification of lens opacities and for DS and control groups.

		DS			Controls			Statistical comparison between groups
		Mean (+/−SD)	Median (IQR)	Range	Mean (+/−SD)	Median (IQR)	Range	
LOCS III grading	Nuclear Opalescence (NO)	0.50 (+/−0.49)	0.40 (0.40)	0.1 to 2.2	1.83 (+/−0.34)	1.80 (0.40)	1.2 to 2.7	z = −7.4, p < 0.00001
	Nuclear Colour (NC)	0.48 (+/−0.58)	0.30 (0.40)	0.1 to 2.5	1.69 (+/−0.45)	1.75 (0.70)	0.5 to 2.5	z = −7.1, p < 0.00001
	Cortical (C)	0.75 (+/−0.71)	0.50 (0.70)	0.1 to 2.4	0.17 (+/−0.37)	0.10 (0.00)	0.1 to 2.6	z = 6.1, p < 0.00001
	Posterior subcapsular (P)	0.38 (+/−0.74)	0.10 (0.00)	0.1 to 2.3	0.10 (+/−0.00)	0.10 (0.00)	0.1 to 0.1	z = 2.8, p < 0.006
AS-OCT quantification of lens opacities	Nuclear PIR	1.19 (+/−0.06)	1.19 (0.7)	1.10 to 1.45	1.25 (+/−0.04)	1.24 (0.06)	1.18 to 1.35	z = −4.8, p < 0.0001
	Cortical PIR	0.09 (+/−0.34)	0.0 (0.0)	0 to 1.46	0.0 (0.0)	0.0 (0.0)	—	
	Cortical PAR	0.006 (+/−0.03)	0.0 (0.0)	0 to 0.15	0.0 (0.0)	0.0 (0.0)	—	
	Posterior subcapsular PIR	0.09 (+/−0.35)	0.0 (0.0)	0 to 1.56	0.0 (0.0)	0.0 (0.0)	—	
	Posterior subcapsular PAR	0.01 (+/−0.05)	0.0 (0.0)	0 to 0.24	0.0 (0.0)	0.0 (0.0)	—	
**Visual Acuity**	0.371 (+/−0.215)	0.300 (0.275)	0.050 to 0.800	−0.053 (+/−0.123)	−0.080 (0.155)	−0.20 to 0.38		

Pixel intensity ratios (PIR) and pixel area ratios (PAR) describe the brightness and extent of any lens changes. Visual Acuity of DS and control participants (LogMAR).

In the DS group, using LOCS IIII photograding, four eyes from four individuals (14.3%) had clinically significant age-related cataracts (n = 1 cortical, n = 2 posterior sub-capsular, n = 1 nuclear sclerotic). These were seen on SL examination, and all were successfully imaged for photograding. Clinically significant cataracts were defined as a LOCS III score^23,24^ of Cortical (C) >/ = 2.0, Posterior sub-capsular (P) >/ = 2.0 and Nuclear Colour (NC) or Nuclear Opalescence (NO) >/ = 2.5. Six control eyes presented with significant nuclear cataract, and cortical cataract was identified in one control eye (n = 7 individuals, 19.4%).

Comparing all LOCS III values, the control group had significantly greater amounts of NO and NC compared to the DS group (z = −7.4, p < 0.00001, and z = −7.1, p < 0.00001 respectively). Conversely, the DS group demonstrated greater magnitude of C and P LOCS III scores compared to controls (z = 6.1, p < 0.00001, and z = 2.8, p < 0.006 respectively).

For the AS-OCT quantification of lens opacities by pixel intensity and pixel area ratios, Table 1 summarises the values for both study groups. Note that none of the control participants displayed quantifiable evidence of posterior sub-capsular or cortical changes. Therefore, statistical analysis was conducted only for nuclear PIR. Participants with DS had significantly lower nuclear PIR compared to controls.

Comparing LOCS III NO and NC grading with AS-OCT quantification of Nuclear PIR there was a significant relation for both DS and control groups (Spearman rank analysis, NO: DS group ρ = 0.36, p = 0.04; Control group ρ = 0.42, p = 0.004. NC: DS group ρ = 0.44, p = 0.01; Control group ρ = 0.43, p = 0.003).

There was a significant relation between age and nuclear PIR for the DS group (ρ = 0.67, p < 0.00001), though this was not apparent for controls (ρ = 0.28, p = 0.06), likely due to the constrained recruitment age.

**Figure 1:**
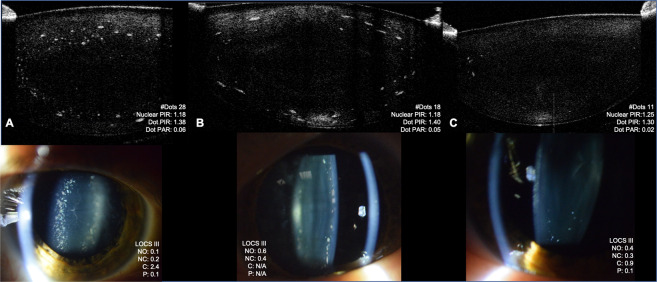
SL and AS-OCT images from three subjects with DS classified with ‘frequent’ (Panel A), ‘moderate’ (Panel B) and ‘few’ (Panel C) dot opacities. Individual LOCS III and PIR/PAR scores are given for each subject. Anterior and posterior lens AS-OCT images were aligned together to present the whole lens.

In the DS group, SL examination and SL and OCT imaging revealed the frequent presence of small dot opacities scattered anteriorly and posteriorly through the cortex of the lens, and occasionally the nucleus. These were distinct from age-related changes. Figure 1 illustrates three subjects classified with ‘frequent’, ‘moderate’ and ‘few’ dot opacities.

There were occasions when assessing DS eyes where dot opacities were noted on SL examination but could not be successfully imaged using either SL or AS-OCT due to difficulties with patient fixation or alignment. Twenty-two lenses had visible dot opacities on either LOCS III or OCT images, with a further five in which the researcher noted the presence of at least one dot opacity on SL examination. Thus, in total, 27 eyes of 54 examined eyes presented with cortical dot opacities (50%) in those with DS. The intensity of these dot opacities was significantly brighter than the intensity of nuclear PIR (p < 0.001), and dot opacities covered an area ranging from 0.2–14% of the cortex (Table 2, OCT image analysis). With regard to location, there was a diffuse spread of dot opacities throughout the cortex, with 53.2% of dots located beyond the central 4 mm of the lens.

**Table 2:** Profile of Dot opacities on OCT imaging of DS lenses (n = 16), graded by Pixel intensity ratio (PIR) and area ratio (PAR).

DS group	Mean (+/−SD)	Median (IQR)	Range
Dot PIR	1.33 (+/−0.12)	1.3 (0.12)	1.14 to 1.68
Dot PAR	0.04 (+/−0.05)	0.02 (0.07)	0.004 to 0.14
Number of dots	15.63 (+/−15.87)	9 (25.25)	1 to 50
Number of dots in central 4–4.5 mm	7.31 (+/−8.05)	2 (13.25)	0 to 23

**Figure 2:**
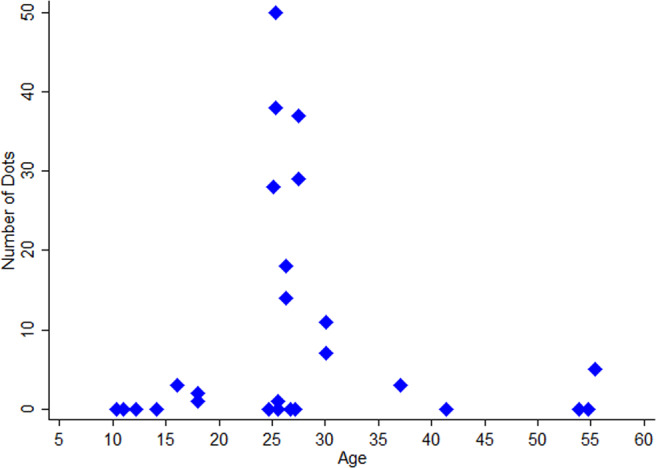
Scatterplot of total number of dot opacities (from AS-OCT imaging) compared with the age profile of participants with DS.

Monocular visual acuity measures with habitual refractive correction were successfully achieved from 94% of the DS group and 100% of control participants (Table 1). Neither Dot PIR nor Dot PAR values were significantly related to DS participant’s visual acuity or age (Mann Whitney, p > 0.5). Figure 2 is a scatterplot of number of dot opacities (from OCT imaging) compared with age of participants with DS.

## Discussion

The current study provides the most detailed evaluation of cataract in Down syndrome to date. Some degree of lens opacity was seen in approximately half (54%) of DS eyes, but in only four individuals (7% eyes, 14% of DS participants) were these opacities classified as clinically significant ‘age-related’ cataract.

Punctate dot opacities, characteristic of developmental cerulean blue-dot type cataracts, were seen in 50% of DS lenses. AS-OCT imaging offers the means of quantifying these deficits, through identification and calculation of the size and pixel intensity of individual opacities. Dot PAR calculation demonstrated that dot opacities covered an area ranging from 0.2–14% of the cortex of the DS lenses in which they were found. The opacities were not detrimental to visual acuity measures, nor were they related to age, but they are intriguing given the possibility that the lens in DS is a potential site for beta-amyloid accumulation.

Goldstein *et al*.^18^ reported, through post-mortem immunohistochemical analysis, that Aβ was present in lenses with a specific type of cataract located in the supranuclear area in individuals with Alzheimer’s disease. This work was developed by Moncaster *et al*.^19^ who reported supranuclear cataract in post-mortem lenses from individuals with DS. Supranuclear cataract is defined as a narrow band of opacity found at the interface between the deep cortex and nuclear interface, and is sometimes referred to as lamellar or zonular^25^. However, the location of the cerulean-type dot opacities found in the present study ranged from close to (and occasionally within) the nucleus to the outer cortex area; they were not confined to the ‘supra-nuclear’ region of the crystalline lens. In this *in-vivo* examination, dot opacities were scattered relatively evenly around the nucleus, with a slightly greater proportion in the periphery. Their presence could easily be missed without pupil dilation. The present investigation found no association between the presence and number of dot opacities in the lens and age of the participant; a finding which is counterintuitive if these dot opacities are indicative of AD (as suggested by Goldstein and colleagues), in a population where prevalence and risk of AD greatly increase with age. Given the findings of the present study and later studies of post-mortem lenses using similar techniques to Goldstein *et al*.^18^ and Moncaster *et al*.^19^ which failed to demonstrate presence of Aβ in the human crystalline lens^26–28^, it seems likely that the relation between cerulean cataract and Aβ found by Goldstein *et al*.^18^ and Moncaster *et al*.^19^ may have been an artefact, reflective of small sample sizes. Further support for the notion that dot opacities are unlikely to be a useful indicator of AD comes from the failure to find supranuclear cataracts in an *in-vivo* analysis of cataract in participants with AD by Bei *et al*.^29^.

The present study found no evidence that individuals with DS have earlier ‘age-related’ nuclear lens changes compared with the typically ageing eye. Only three DS participants had NO or NC grades over 1.0, and they were 55.4, 53.9 and 54.7 years of age. These participants were all in the same age bracket as our control group and their LOCS III nuclear grades fit within the normal range of the control group’s distributions. When comparing AS-OCT nuclear PIR values between the DS and control groups, the results were similar to LOCS III classification, demonstrating significantly lower nuclear PIR in the DS compared to control group.

When compared to the literature describing the prevalence of crystalline lens opacities in DS, the present study identifies opacity in 54% of the 54 eyes: previous studies have reported prevalence ranging from 4–72%^8–13^. Studies reporting low levels of lens opacity were primarily conducted in children and adolescents and this finding chimes with the present study in which all participants under 12 years of age (where imaging was possible, n = 5) had clear lenses.

Considering previous studies of adult DS eyes, our findings are similar to those reported by Jaeger^8^ who undertook SL examination of the eyes of 74 participants with DS aged 15 to 64 years, and found 55.4% to have the presence of lens opacities. Of these opacities, the majority (65.8%) were described as ‘flake-like’ opacities, with 18.9% of DS participants (average age 48 years) exhibiting age-related cataract^8^. More recently Fong *et at*.^10^ examined 91 adults with DS aged from 30 to 56 years, and report that 72% of participants presented with lens opacities. Of those opacities, 50.6% were described as blue-dot cataract and 45% described as ‘age-related’ (38% nuclear, 13.6% cortical and 8.5% PSC). These prevalence rates align quite well with those found in this study.

The flake-like and blue-dot opacities that are reported at high prevalence by Jaeger^8^ and Fong *et al*.^10^ appear to be of the same morphology as the dot opacities imaged in this study. Cerulean blue-dot cataract is an acquired early onset lens opacity that is often noticed first in adolescence and consists of blue or white coloured opacities scattered primarily in the cortex but also in the nucleus of the crystalline lens^30,31^.

AS-OCT imaging offers a useful way to image the crystalline lens in DS and was a more successful method to capture images of the lens than SL photography. Furthermore, the OCT images have provided a unique insight into the typical location of cerulean blue-dot opacity within the DS crystalline lens. As a result, the present study has shown that cerulean cataract occurs commonly in DS, but the location and morphology of this cataract does not appear to coincide with the description by studies linking it with Aβ and AD.

In common with other studies recruiting individuals with Down syndrome, it is difficult to generate a large sample size, and reduced success rates in acquiring adequate quality images of the crystalline lens. However, the study was successful in recruiting across a broad age range and older participants with DS. Advances in OCT technology, especially with the newest swept-source whole eye imaging capability, will provide an excellent platform for studying opacity in the entire lens, enabling quantification of opacities with greater precision. Further work investigating cataract in Down syndrome in longitudinal studies would be valuable to understand the development of cerulean blue-dot cataracts and what, if any, relevance their presence or absence has for the individual with Down syndrome.

## Methods

The study received ethical approval from the NHS Office for Research Ethics Committees in Northern Ireland and Wales, UK. All methods were carried out in accordance with relevant guidelines and regulations. Thirty participants with Down syndrome between the ages of 6 and 60 years were recruited from regional day centres, family support groups, and the optometry clinics of Ulster and Cardiff Universities. Parental consent was obtained for children to participate and either the adult with DS consented or if this was not possible, their assent was recorded and written consent obtained from their carer. Exclusion criteria were previous cataract surgery, nystagmus, ocular albinism, keratoconus, and other significant corneal pathology that would affect imaging of the crystalline lens. A group of adults aged 50–60 years acted as a control group. This age range was chosen to uncover age-related lens changes, but not to over-represent cataract liable to be found in adults >60 years: from prior literature, the authors considered this to be reflective of the likely status of the DS lens. 36 participants (56 eyes) were recruited through the local population and staff at Ulster and Cardiff Universities.

Participants visited the Centre for Optometry and Vision Sciences at Ulster and Cardiff Universities on one occasion for assessment with a single researcher, who was also a registered optometrist. Clinical history was taken to ensure inclusion and exclusion criteria were met. Referral to the appropriate care provider was organised if clinically significant ocular findings were revealed in the course of examination. Visual acuity was measured prior to dilation (with habitual refractive correction worn) using Keeler crowded LogMAR letter charts, or the crowded Lea symbols if letter optotype testing was not possible. Pupil dilation was achieved with 1.0% tropicamide and intraocular pressure was measured with the ICare tonometer (ICare, Finland) pre- and post-dilation as a significant increase would indicate an adverse event of angle-closure glaucoma requiring medical intervention. Pupil diameter was also measured after maximal dilation had occurred. Crystalline lens images were obtained using a Nikon D3000 camera mounted onto a Nikon FS-3 Slit lamp (SL) and a Zeiss Visante anterior segment OCT (Carl Zeiss Meditec) in that order.

Crystalline lens examination and imaging was undertaken in a dark room using the modified Nikon FS-3 slit-lamp: attempts were made to capture multiple optic sections and retroillumination images attempted for all crystalline lenses. Camera and illumination settings were kept constant and in accordance with LOCS III protocol. When imaging cortical and PSC opacities, slit lamp settings were varied to obtain optimum retroillumination, again, in accordance with LOCS III protocol.

LOCS III photograding of SL images was conducted for Nuclear Opalescence (NO), nuclear colour (NC), cortical (C) and posterior sub-capsular (P) cataract types. After training on LOCS III system, photograding was conducted by a single examiner (AM). Agreement in scoring was evaluated for 10% of images with a second examiner (JAL) and revealed excellent agreement, with less than a 0.1 LOCS III score of bias for all measures of opacification. Images were graded using the same computer terminal and monitor with uniform brightness and colour settings, and a lightbox placed beside monitor was used for LOCS III transparencies. A grade between 0.1 (representing complete nuclear transparency) and 6.9 was assigned for NO and NC. In accordance with instructions, care was taken to grade NO based strictly by examining the hazy quality of the nucleus without respect to its brunescence. For NC evaluation, the haziness of the nucleus was ignored and a grade was assigned based upon the brunescence reflected from of the posterior capsule. For C and P opacities, the grader considered all areas of opacification solely as an aggregate to determine values for C and P ranging from 0.1 to 5.9. Opacity was considered to be a true PSC only if it was located in the central 3 mm of the lens.

Visante AS-OCT lens images were captured in raw image high-resolution mode, and separate B-Scans of the anterior and posterior lens were acquired. Alignment was determined with the corneal reflex and B-scans were taken at a 90-degree orientation. If participants’ lids interfered with imaging due to narrow palpebral aperture, 180-degree B-scans were also acquired. If necessary, additional B-scans were captured at any other axial orientation in order to image lens opacities observed during slit-lamp imaging; this ensured all lens opacities were captured with AS-OCT imaging. Finally, an image of the whole lens was also captured with the OCT instrument’s low-resolution mode where possible.

**Figure 3:**
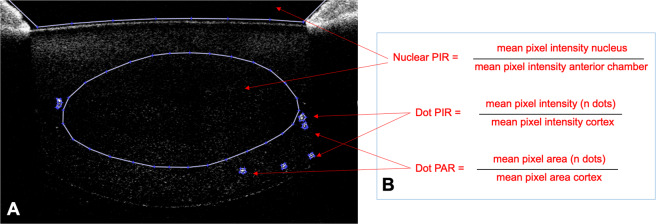
Panel (A) AS-OCT Image (low-resolution) showing segmentation of nucleus, anterior chamber and individual dot opacities. Panel (B) formulae to calculate Nuclear PIR and Dot PIR and PAR.

Raw OCT images were processed in MATLAB (MathWorks Inc., USA) and opacities analysed by calculating pixel intensity ratios (PIR) for nuclear lens areas, and pixel intensity and area ratios (PAR) for cortex and PSC lens areas (see Fig. 3).

Raw detector data were exported for all scans using the Visante Image Exporter software (Carl Zeiss Meditec, Germany). The sensor data were then processed, segmented and analysed for opacity with MATLAB using customised, bespoke software. The image was read as a 512 × 512 matrix and resized to the manufacturer’s recommended 512 × 1280 resolution using bicubic interpolation as described by Kao *et al*.^32^ Segmentation of the lens was undertaken manually by AM. When analysing nuclear opacities, the nucleus was segmented from the anterior and posterior images of each half of the lens: the mean pixel intensity of the nucleus was then compared to that of the aqueous humour (to represent background intensity level) with their ratio representing nuclear PIR. Cortical lens opacities were also segmented and their mean pixel intensity compared as a ratio with the background mean pixel intensity of the entire opacity-free cortex, representing cortical PIR. The area of the cortical opacities was also calculated as a ratio of their mean area compared to the area of the cortex. The PIR and PAR of posterior-subcapsular opacities were calculated in a similar manner to cortical opacities. In the DS eyes, dot opacities (see Fig. 1), were also visualised. These were individually identified and in addition to count, Dot PIR and Dot PAR were calculated. Finally, the central portion of the B-scan, covering the central 4–4.5 mm of the lens, was segmented in MATLAB and the proportion of dots in the central area versus the periphery was calculated.

Statistical analysis was conducted in Stata. Kruskal-Wallis rank sum test was used for comparison between groups as LOCS III data was non-parametric. Pixel intensity ratios were normally distributed for the control group, and one-away ANOVA compared group differences. To examine association, linear regression and Spearman rank (ρ) analysis were used, depending on normality of data.

## References

[1] Bell R, Rankin J, Donaldson LJ (2003). Northern congenital abnormality survey steering group. Down’s syndrome: occurrence and outcome in the north of England, 1985–99. Paediatr. Perinat. Epidemiol..

[2] Haugen OH, Høvding G (2001). Strabismus and binocular function in children with Down’s syndrome. A population-based, longitudinal study. Acta Ophthalmol. Scand..

[3] Little J-A, Woodhouse JM, Lauritzen JS, Saunders KJ (2007). The impact of optical factors on resolution acuity in children with Down’s syndrome. Invest. Ophthalmol. Vis. Sci..

[4] Little J-A, Woodhouse JM, Saunders KJ (2009). Corneal power and astigmatism in Down syndrome. Optom. Vis. Sci..

[5] Ljubic A, Trajkovski V, Stankovic B (2011). Strabismus, refractive errors and nystagmus in children and young adults with Down’s syndrome. Ophthalmic Genet..

[6] Doyle L, Saunders KJ, Little J-A (2016). Trying to see, failing to focus: near visual impairment in Down syndrome. Sci. Rep..

[7] Catalano RA (1990). Down syndrome. Surv Ophthalmol..

[8] Jaeger EA (1980). Ocular findings in Down’s syndrome. Trans. Am. Ophthalmol. Soc.

[9] Liza-Sharmini AT, Azlan ZN, Zilfalil BA (2006). Ocular findings in Malaysian children with Down syndrome. Singapore Med. J..

[10] Fong AHC (2013). Prevalence of ocular abnormalities in adults with Down syndrome in Hong Kong. Br. J. Ophthalmol..

[11] Wong V, Ho D (1997). Ocular abnormalities in Down syndrome: An analysis of 140 Chinese children. Pediatr. Neurol..

[12] da Cunha RP, Moreira JBD (1996). Ocular findings in Down’s syndrome. Am. J. Ophthalmol.

[13] Kim JH, Hwang JM, Kim HJ, Yu YS (2002). Characteristic ocular findings in Asian children with Down syndrome. Eye..

[14] Bittles AH, Bower C, Hussain R, Glasson EJ (2007). The four ages of Down syndrome. Eur. J. Public Health.

[15] Haargaard B, Wohlfahrt J, Fledelius HC, Rosenberg T, Melbye M (2004). A nationwide Danish study of 1027 cases of congenital/infantile cataracts: etiological and clinical classifications. Ophthalmology..

[16] Zana M, Janka Z, Kálmán J (2007). Oxidative stress: A bridge between Down’s syndrome and Alzheimer’s disease. Neurobiol. Aging..

[17] Lee N-C, Chien Y-H, Hwu W-L (2017). A review of biomarkers for Alzheimer’s disease in Down syndrome. Neurol Ther..

[18] Goldstein, L. E. *et al*. Cytosolic β-amyloid deposition and supranuclear cataracts in lenses from people with Alzheimer’s disease. *Lancet.* **361**, 1258–1265 (2003). 10.1016/S0140-6736(03)12981-9 12699953

[19] Moncaster, J. A. *et al*. Alzheimer’s disease amyloid-beta links lens and brain pathology in Down syndrome. *Plos one.* **20**, e10659, 10.1371/journal.pone.0010659 (2010). 10.1371/journal.pone.0010659 PMC2873949 20502642

[20] de Castro A (2018). Three-dimensional cataract crystalline lens imaging with swept-source optical coherence tomography. Invest. Ophthalmol. Vis. Sci..

[21] Grulkowski S (2018). Volumetric macro- and micro-scale assessment of crystalline lens opacities in cataract patients using long-depth-range swept source optical coherence tomography. Biomed. Opt. Express..

[22] Chen D (2018). Lens nuclear opacity quantitation with long-range swept-source optical coherence tomography: correlation to LOCS III and a scheimpflug imaging-based grading system. Br. J. Ophthalmol..

[23] Tan AC (2011). Cataract prevalence varies substantially with assessment systems: comparison of clinical and photographic grading in a population-based study. Ophthalmic Epidemiol..

[24] Mann AL, Bressler SB, Hawkins BS, Holekamp N, Bressler NM (2008). Submacular surgery trials research group. Comparison of methods to identify incident cataract in eyes of patients with neovascular maculopathy: Submacular surgery trials report No. 18. Ophthalmology..

[25] Chylack LT, Lee MR, Tung WH, Cheng HM (1983). Classification of human senile cataractous change by the American cooperative cataract research group (CCRG) Method: 1. Instrumentation and Technique. Invest. Ophthalmol. Vis. Sci..

[26] Michael R (2013). Absence of beta-amyloid in cortical cataracts of donors with and without Alzheimer’s disease. Exp. Eye Res..

[27] Ho CY, Troncoso JC, Knox D, Stark W, Eberhart CG (2014). Beta-amyloid, phospho-tau and alpha-synuclein deposits similar to those in the brain are not identified in the eyes of Alzheimer’s and Parkinson’s disease patients. Brain Pathol..

[28] Michael R (2014). Absence of amyloid-beta in lenses of Alzheimer patients: A confocal Raman microspectroscopic study. Exp. Eye Res..

[29] Bei L (2015). A test of lens opacity as an indicator of preclinical Alzheimer disease. Exp. Eye Res..

[30] Armitage MM, Kivlin JD, Ferrell RE (1995). A progressive early onset cataract gene maps to human chromosome 17q24. Nat. Genet..

[31] Kanski, J. J. & Bowling, B. *Clinical Ophthalmology: A Systematic Approach*. 6th Edition. Expert Consult. Edinburgh; New York: Elsevier/Saunders (2011).

[32] Kao C-Y, Richdale K, Sinnott LT, Grillott LE, Bailey MD (2011). Semiautomatic extraction algorithm for images of the ciliary muscle. Optom. Vis. Sci..

